# Temporal and Spatial Patterns of Glial Activation After Unilateral Cortical Injury in Rats

**DOI:** 10.3390/life16010142

**Published:** 2026-01-15

**Authors:** Karen Kalhøj Rich, Simone Hjæresen, Marlene Storm Andersen, Louise Bjørnager Hansen, Ali Salh Mohammad, Nilukshi Gopinathan, Tobias Christian Mogensen, Åsa Fex Svenningsen, Mengliang Zhang

**Affiliations:** 1 Department of Molecular Medicine, University of Southern Denmark, DK-5230 Odense, Denmark; krich@health.sdu.dk (K.K.R.); shjaresen@health.sdu.dk (S.H.); stormandersen@sdu.dk (M.S.A.); lobhansen@health.sdu.dk (L.B.H.); almoh19@student.sdu.dk (A.S.M.); nigo@bio.aau.dk (N.G.); tcmogensen@health.sdu.dk (T.C.M.); aasvenningsen@health.sdu.dk (Å.F.S.); 2 Department of Physics, Chemistry and Pharmacy, University of Southern Denmark, DK-5230 Odense, Denmark; 3 Brain Research-Inter Disciplinary Guided Excellence (BRIDGE), University of Southern Denmark, DK-5230 Odense, Denmark

**Keywords:** traumatic brain injury, neuroinflammation, microglia activation, astrocytic gliosis, motor deficits, focal cortical injury, glia-neuronal interactions, rat model

## Abstract

Traumatic brain injury (TBI) often leads to long-lasting motor deficits, but the underlying cellular mechanisms still remain poorly understood. In this study, we examined glial and neuronal responses after focal cortical aspiration injury of the right hindlimb sensorimotor cortex in adult male rats. This is a model we have previously shown induces persistent gait asymmetry and postural deficits. Immunohistochemical analysis of activated microglia/macrophages (CD11b, IBA-1), astrocytes (GFAP), and neurons (NeuN) was performed bilaterally in the peri-lesional cortex at 3, 7, 14, 21, and 28 days post-injury (*n* = 3–6 per time point). The injury induced an early, sharply localized increase in CD11b-positive myeloid cells in the injured hemisphere, suggesting an activation of both resident microglia and infiltrating monocyte-derived cell. This was followed by a more sustained IBA-1-positive microglial activation that gradually extended contralaterally. Astrocytic activation showed a delayed but prolonged profile, rising ipsilaterally within the first week, peaking around two weeks, and becoming bilaterally elevated by four weeks. Sham-operated animals showed only basal glial immunoreactivity without signs of hypertrophy or reactive morphology at any time point. NeuN immunoreactivity remained stable across timepoints, suggesting preservation of neuronal soma labeling without evidence of overt secondary neuronal loss. These findings reveal a staged and spatially distinct glial response to focal cortical injury, with early myeloid activation, prolonged microglial reactivity, and delayed bilateral astrogliosis. Together, these findings are consistent with the possibility that persistent motor deficits after focal TBI arise from both primary tissue loss within the lesion core and peri-lesional glial remodeling, highlighting glial–neuronal interactions as a potential therapeutic target.

## 1. Introduction

Traumatic brain injury (TBI) results from external mechanical forces and can lead to temporary or permanent impairments in physical, cognitive, and psychosocial functions [1]. It is a major global health problem, with estimated 64–74 million people sustaining a TBI each year, most commonly due to road traffic injuries and falls. Incidence is particularly high in adolescents, young adults and in older adults [2].

The pathology of TBI is commonly divided into two phases. The primary injury reflects the immediate mechanical disruption of brain tissue, whereas the secondary injury develops over hours to weeks and involves neuroinflammation, blood–brain barrier (BBB) disruption, edema, and excitotoxicity [3]. These processes expand the lesion and worsen neuronal dysfunction. Persistent or excessive inflammation has been linked to progressive neuronal loss and lasting functional deficits [4]. Outcomes are also influenced by injury location, as focal cortical injuries are often associated with long-lasting contralateral motor impairments, including spastic paresis, gait disturbances, and deficits in fine motor control [5].

Recently, we showed that a unilateral lesion of the hindlimb sensorimotor cortex in rats induced robust and persistent motor deficits, including contralateral hindlimb postural asymmetry (HL-PA) and gait alterations [6]. These findings provided a functional basis for investigating whether lateralized neuroinflammatory responses develop in parallel with motor asymmetries.

The immune response to TBI is initiated when damage-associated molecular patterns (DAMPs) activate pattern recognition receptors on glial cells, triggering inflammatory cascades involving nuclear factor kappa-light-chain-enhancer of activated B cells (NF-κβ) signaling and cytokine release [3,4]. Microglia are the first responders and can adopt pro- or anti-inflammatory phenotypes. In their pro-inflammatory state, they release interleukins like IL-6 and IL-1β, tumor necrosis factor alpha (TNF-α), glutamate, and reactive oxygen species, thereby aggravating neuronal stress. In their anti-inflammatory state, they contribute to repair through phagocytosis and the release of IL-4 and IL-10. Infiltrating macrophages can also influence these processes, although their role in focal cortical injury remains poorly understood [3,4,7]. Astrocytes are activated early as well and may assume neurotoxic or neuroprotective functions. They support BBB integrity, release trophic factors, and stabilize the extracellular environment, but persistent activation can prolong inflammation and restrict recovery through scar formation [3,4,7].

Rodent models of TBI differ in the distribution of injury and in their inflammatory and motor outcomes. Weight-drop and fluid percussion injury (FPI) models generally produce diffuse pathology, often affecting both cortical and subcortical regions, as well as white matter tracts. These injuries are accompanied by widespread gliosis and by time- and region-dependent variations in microglial and astrocytic response. For example, the degree of glial fibrillary acidic protein (GFAP) reactivity after FPI depends strongly on the location of the craniotomy [7,8]. Because injury and gliosis are often diffuse, inflammation is often bilateral or global, resulting in less consistently lateralized motor impairments.

In contrast, controlled cortical impact (CCI) and aspiration/ablation models induce focal cortical lesions with reproducible tissue responses concentrated in peri-lesional regions [7,8]. These focal injuries are typically associated with predictable contralateral motor impairments, including hemiparesis, gait asymmetry, and HL-PA, reflecting the decussation of corticospinal pathways. This allows direct links to be made between local neuroinflammatory responses and lateralized motor outcomes. In our aspiration model targeting the hindlimb sensorimotor cortex, we previously observed persistent contralateral HL-PA and altered gait, along with evidence that these asymmetries are encoded at the spinal level [9]. These findings highlight the value of focal TBI models for investigating how cortical injury drives neuroinflammation and asymmetric motor deficits.

In this study, we examined glial and neuronal responses to focal TBI in the rat hindlimb sensorimotor cortex. Our aim was to determine whether inflammatory and cellular changes differ between the ipsilateral and contralateral hemispheres, and how these patterns relate to the motor outcomes we previously reported in the same rats [6]. While unilateral cortical injury is generally expected to produce contralateral motor impairments due to corticospinal decussation, our earlier work showed that aspiration of the hindlimb sensorimotor cortex induces persistent postural asymmetry and gait alterations that cannot be fully explained by a simple ipsi–contra distinction. This raises the question of whether glial activation also extends beyond the lesioned hemisphere. We therefore hypothesized that focal TBI would elicit a sequential neuroinflammatory response, beginning with an acute ipsilateral cluster of differentiation 11b (CD11b) microglial/macrophage component, followed by more sustained ionized calcium-binding adapter molecule 1 (IBA-1) microglial activation and delayed astrocytic GFAP upregulation, and that these glial changes could extend contralaterally in parallel with the evolution of motor deficits.

## 2. Materials and Methods

### 2.1. Animals

Adult male Sprague-Dawley rats (Janvier Labs, weight 250–600 g, 3–4 months) were housed under standard laboratory conditions (12 h light/dark cycle, 21 °C, 65% humidity) with ad libitum access to food and water. Animals were randomly assigned either the TBI group or a SHAM-operated control group. All experimental procedures were conducted in accordance with institutional and national ethical guidelines (Permit # 2019-15-0201-0015).

### 2.2. Focal Cortical Aspiration Model

Focal TBI was induced in the right hindlimb sensorimotor cortex using a focal cortical aspiration model, as previously described. The animals used in the present study are the same cohort as those reported in the previous behavioral study [6]. The rats were given oral buprenorphine (Temgesic^®^ 485473, Indivior Europe, Richmond, VA, USA) 0.4 mg/kg 1 h pre-operatively mixing the drug with peanut butter; the drug acts over 24 h and was used for postoperative pain relief.

The rats were anesthetized intraperitoneally (i.p.) with a mixture of ketamine 100 mg/kg (Ketaminol Vet 20 mg/mL, CAS: 6740-88-1, MSD Animal Health, Stockholm, Sweden) and xylazine 10 mg/kg (Rompun Vet 50 mg/mL, CAS: 7361-61-7, Elanco Denmark, Ballerup, Denmark) with a dose of 0.25 mL/kg body weight. To avoid eye irritation during surgery, 2 mg/g carbomer gel (ViscoTears, CAS: 9007-20-9, Bausch & Lomb Nordic AB, Stockholm, Sweden) was applied to the eyes.

Local anesthesia of 20 mg/mL lidocaine (Xylocain, CAS: 137-58-6, Aspen Nordic, Aspen, CO, USA) was given in the ears and subcutaneously on the surgical area. The rats’ heads were fixed on a stereotaxic head holder to ensure proper alignment of bregma and lambda landmarks. Under local anesthesia with lidocaine, the scalps were incised along the midline, opened, and stretched to expose the right side of the cranium.

A section of the cranium was marked with coordinates 0.5–4.0 mm posterior to bregma and 1.8–3.8 mm lateral to the midline. The marked cranial area was opened by drilling. The part of the cerebral cortex located below this window includes the hindlimb representation area of the sensorimotor cortex, which was aspirated with a glass pipette (tip diameter ~0.5 mm) connected to an electrical suction machine (Craft Duo-Vec Suction unit, Rocket Medical Plc, Watford, UK). The craniotomy measured approximately 3.5 × 2 mm. As previously documented for these animals, histological assessment confirmed that the resulting cortical lesion was confined to the cortex and did not extend into the underlying white matter [6]. The lesion extended approximately 3.6–4.0 mm rostrocaudally, 2.4–3.0 mm mediolaterally, and 1.3–1.7 mm in depth, corresponding to a lesion volume of 6.2 ± 0.8 mm^3^ after correction for tissue shrinkage due to fixation [6]. The lesion was sharply demarcated, allowing reliable identification of the lesion margin in all animals and at all time points. Care was taken not to aspirate the white matter below the cortex. After suction was complete, bleeding was stopped using Spongostone (Lagaay Medical, Rotterdam, The Netherlands). The wound was closed using 3-0 vicryl suture (Ethicon, Raritan, NJ, USA), and lidocaine was reapplied on the operation area.

After the operation, the rats were placed in a recovery cage for 24 h and then returned to their home cage.

For the SHAM operation, the same anesthetic and operative procedure was used, but the dura was kept intact, and no cortical tissue was aspirated. After hemostasis, the wound was closed in the same manner as in the TBI group.

This aspiration model produces focal cortical lesions that consistently result in contralateral motor deficits, including HL-PA and flexion, thereby providing a reproducible phenotype for linking motor impairments with tissue responses [9].

### 2.3. Perfusion and Tissue Preparation

Animals were euthanized on days 3, 7, 14, 21, and 28 post-injury. Following the completion of HL-PA and gait pattern assessments [6], the rats were administered a lethal dose of sodium pentobarbital (60 mg/100 g body weight, Exagon Vet., Salfarm, CAS: 76-74-4) via i.p. injection. Subsequently, intracardiac perfusion fixation was performed. The perfusion procedure began with 0.1 M phosphate-buffered saline (PBS) followed by a fixative solution containing 4% paraformaldehyde (Sigma-Aldrich/Merck, Darmstadt, Germany, CAS: 30525-89-4) in 0.1 M PBS at a volume of 100 mL/100 g body weight. After perfusion, the brain was carefully dissected and post-fixed in 4% paraformaldehyde at 4 °C for 24 h and cryoprotected in 30% sucrose with 0.1% sodium azide (NaN_3_). Tissue was stored at −80 °C until being sectioned coronally at 40 µm using a freezing microtome (Microm 34, Thermo Fisher Scientific, Roskilde, Denmark). The sections were distributed into ten separate Eppendorf tubes, with every 10th section placed in the same tube to ensure uniform sampling. The storage solution in each tube contained 30% sucrose and 0.1% NaN_3_, which prevented crystal formation in the tissue during storage.

### 2.4. Immunohistochemistry (IHC) Protocol

Coronal brain sections from each animal were stained for one of four cellular markers: CD11b identifying microglia and infiltrating monocytes/macrophages [10], IBA-1 labeling the total microglia/macrophage population [11,12], GFAP detecting astrocytes [13,14], and neuronal nuclei (NeuN) identifying mature neurons [15]. CD11b is a membrane integrin associated with adhesion, phagocytosis, and cytokine release [10]. IBA-1 is a cytoplasmic calcium-binding protein linked to actin remodeling and migration, and its expression increases with microglial activation [11,12]. GFAP labels intermediate filaments in astrocytes, which respond to injury through processes including BBB repair, neurotrophic support, and glial scar formation [13,14]. NeuN serves as a neuronal reference marker, allowing evaluation of glial changes relative to neuronal density [15].

Endogenous peroxidase activity was quenched for 30 min (0.3% H_2_O_2_ in PBS), and non-specific binding was blocked in 2% bovine serum albumin (BSA) and 5% normal goat serum (NGS) for 1 h. Primary antibodies (diluted in 5% goat serum, 2% BSA and PBS with 0.1% triton X-100 (PBS-T) were applied for 20 min at room temperature (RT) followed by approx. 48 h at 4 °C (for further information on the antibodies and their concentrations see Supplementary Table S1).

After primary antibody incubation, sections were brought to RT for 30 min and washed in PBS-T three times for 15 min. The sections were incubated with biotinylated secondary antibodies in PBS-T containing 1% BSA and 2% NGS for 2 h at room temperature followed by incubation in an avidin–biotin complex (ABC) solution (1:100; Vectastain Elite ABC kit, peroxidase standard (Vector Laboratories, Burlingame, CA, USA) in PBS-T) for 1 h at RT (for further information on the antibodies and their concentrations, see Supplementary Table S1). After incubation, sections were washed in PBS for two 10 min washes, followed by two 10 min washes in tris-buffered saline (TBS) to remove any excess avidin. The chromogenic development was carried out by incubating sections in TBS (pH 7.2) containing 0.05% 3,3′-diaminobenzidine (DAB) (Merck, D5637, Hessen, Germany) and 0.01% H_2_O_2_ until the desired brown coloration developed, which typically took 1–15 min depending on the primary antibody used. Sections were then washed in TBS five times for 5 min each. The sections were then washed in tris buffer and mounted onto glass slides using a 0.5% gelatine solution.

Once dry, the sections were dehydrated in graded alcohol (70%, 96%, and 99.9%) followed by xylene and mounted with DPX mounting medium (Merck, HX60964379, Burlington, VT, USA).

### 2.5. Data Acquisition and Analysis

**Figure 1:**
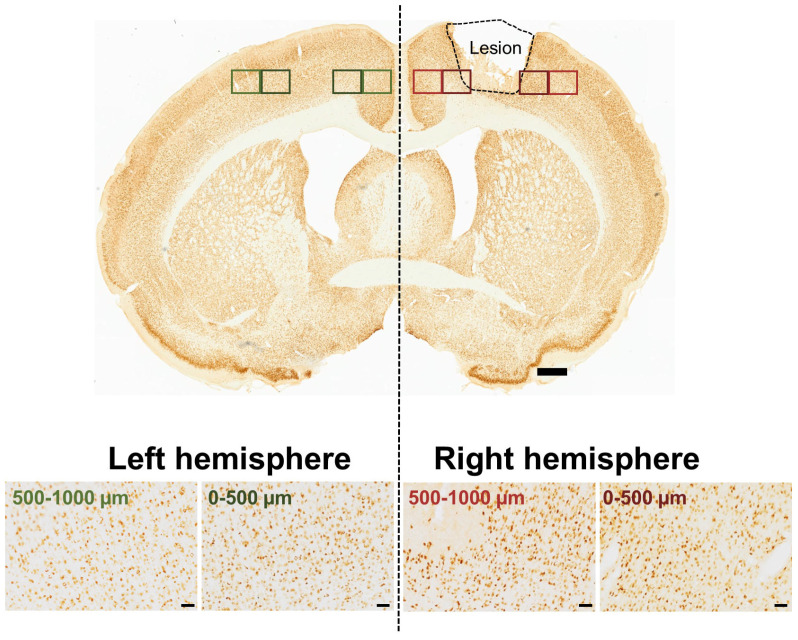
Standardized regions of interest. Representative NeuN-stained section from a TBI rat at day 7 post-injury illustrating the standardized regions of interest for quantification in the TBI-affected (**right**) and contralateral (**left**) sensorimotor cortex. Red boxes indicate sampling sites in the lesioned hemisphere (0–500 µm from the lesion edge: dark red; 500–1000 µm: light red), while green boxes indicate contralateral sites (0–500 µm: dark green; 500–1000 µm: light green). Scale bars: 500 µm (overview) and 50 µm (insets).

The brain sections were imaged using a LEICA DM6000 B light microscope (Leica Microsystems, Wetzlar, Germany) at 20× magnification in 8-bit grayscale mode. A total of 40 rat brains were analyzed, comprising 22 with TBI and 18 SHAM-operated controls (see Supplementary Table S3). For each brain, four coronal sections were imaged (with a Leica DFC420 digital microscope camera, Digital Camera System, Wetzlar, Germany) per marker (CD11b, IBA-1, GFAP, or NeuN), and eight images were captured per section—two each from regions located 0–500 µm and 500–1000 µm lateral to the lesion site in both hemispheres (see Figure 1). In TBI animals, regions of interest were defined relative to the visible lesion margin in each section, rather than based on absolute stereotaxic coordinates. This approach ensured consistent peri-lesional sampling across animals despite potential inter-individual variability in lesion extent. In SHAM animals, comparable midline-symmetric regions were imaged, so identical sampling zones were used across hemispheres and timepoints to assess spatial and lateralized glial reactivity. Exposure and acquisition settings were kept identical across all samples for a given marker to allow valid comparison.

**Figure 2:**
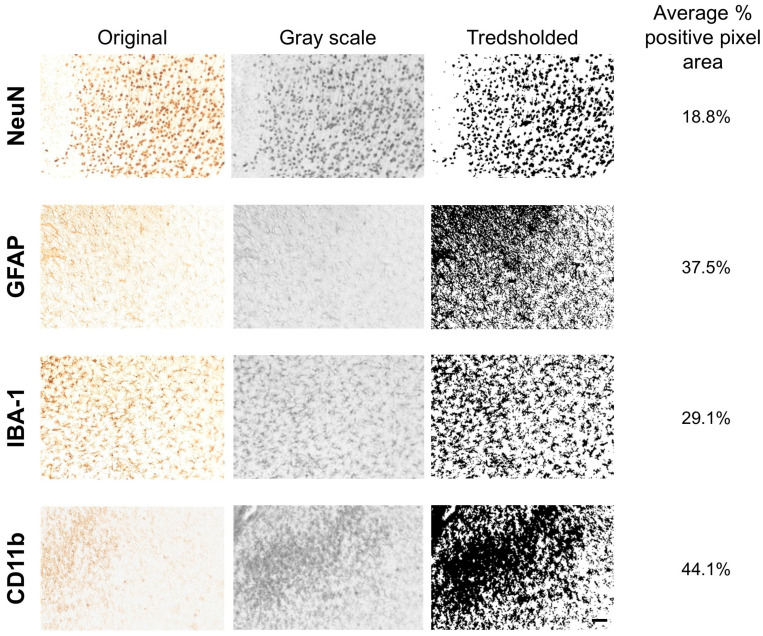
Image processing workflow used for quantification of immunohistochemical staining. Representative images from TBI rats at day 7 post-injury are shown for each marker (NeuN, IBA-1, CD11b, GFAP) to illustrate the analysis pipeline. The first column shows the original DAB-stained image, the second column the grayscale-converted version used for thresholding, the third column the thresholded binary image used for quantification (with a fixed threshold per marker), and the fourth column the calculated percent area of positive staining within the region of interest (average), as extracted using ImageJ (Fiji). Scale bar, valid for all panels, 50 µm.

Quantification of immunoreactivity was performed using Fiji (ImageJ 1.54g, Java 1.8.0_364 (64.bit)). A custom batch-processing macro was applied for most datasets. This pipeline included background subtraction (rolling ball radius), application of a fixed grayscale threshold, and measurement of the percent area of positive staining (see Figure 2 and Supplementary Table S2). For each marker and timepoint, threshold values were held constant to ensure comparability.

For CD11b, all timepoints were quantified using the macro. For IBA-1, GFAP, and NeuN, all timepoints except day 28 were analyzed using the macro. Day 28 images were quantified manually by a different researcher using the same ImageJ processing steps (background subtraction, thresholding, and percent area measurement), but with threshold values selected manually rather than applied via the batch macro. Additionally, while day 3 and day 14 images for IBA-1, GFAP, and NeuN were immunostained by different researchers, their quantification was performed using the macro pipeline.

To account for potential variability related to staining intensity, imaging conditions, or threshold selection, all values were normalized to time-matched SHAM controls prior to statistical analysis. This normalization ensured that reported values reflect relative differences between experimental conditions rather than absolute staining intensity.

For each brain, data from 3 sections per hemisphere were averaged to produce one representative value per region and marker. All stained brain sections that were imaged were included in the analysis; although the number of animals analyzed differed between markers, no imaged sections or images were excluded.

### 2.6. Statistical Analyses

All statistical analyses were performed using GraphPad Prism (version 9.5.1, GraphPad Software, LLC, Boston, MA, USA). Due to batch variability between staining sessions, all expression values were normalized to the average of SHAM left + SHAM right values at the corresponding timepoint and distance.

As a result, normalized values reflect relative differences compared to time-matched SHAM controls and are not suitable for direct comparisons across different timepoints. Variability in the SHAM signal between experimental days can therefore lead to substantial differences in fold-change values across timepoints, independent of absolute changes in TBI immunoreactivity. This approach controls for variability in staining or imaging conditions between sessions, but absolute expression levels should not be interpreted longitudinally. All statistical comparisons were therefore restricted to within-timepoint contrasts (e.g., TBI vs. SHAM; ipsilateral vs. contralateral).

Normality of the data was evaluated by testing the residuals from each two-way repeated-measures ANOVA model using the Shapiro–Wilk test. In addition, visual inspection of QQ-plots of the original datasets showed no substantial deviations from a normal distribution. All residuals passed the Shapiro–Wilk test (*p* > 0.05), indicating that the ANOVA normality assumption was met.

For each marker and timepoint, group differences between TBI and SHAM animals were analyzed using a two-way ANOVA mixed-effects model (group as between-subject factor, hemisphere as within-subject factor). When significant main or interaction effects were detected, hemisphere-specific post hoc comparisons (SHAM left vs. TBI left; SHAM right vs. TBI right) were performed. Multiple comparisons were controlled using the Holm–Sidak method, which offers strong family-wise error control while being less conservative than the classical Bonferroni correction. The significance level for these analyses was set at *p* < 0.05.

Lesion lateralization within TBI animals (right vs. left hemisphere) was assessed using paired *t*-tests. As multiple paired comparisons were carried out across timepoints and distances, Holm–Sidak correction was manually applied within each family of tests, resulting in an adjusted significance threshold of *p* < 0.0253.

All data are reported as mean ± SD.

## 3. Results

To investigate the cellular responses to focal cortical injury, we quantified immunoreactivity for markers of microglia/macrophages, astrocytes, and neurons across time and space (the number of animals in each group used for the analyses is stated in Supplementary Table S3). Below, we report on the temporal dynamics and hemispheric distribution of these markers. Because all expression values were normalized to time-matched SHAM controls, absolute values cannot be directly compared across timepoints. Group comparisons were therefore restricted to within each timepoint.

### 3.1. CD11b Expression Reveals Early, Sharply Lateralized Microglial/Macrophage Activation That Resolves by Day 21

We first examined CD11b expression to assess early microglial/macrophage response following TBI. This marker is known to highlight reactive microglia and potentially infiltrates myeloid cells [10]. CD11b revealed a rapid, sharply lateralized response that resolved within three weeks.

**Figure 3:**
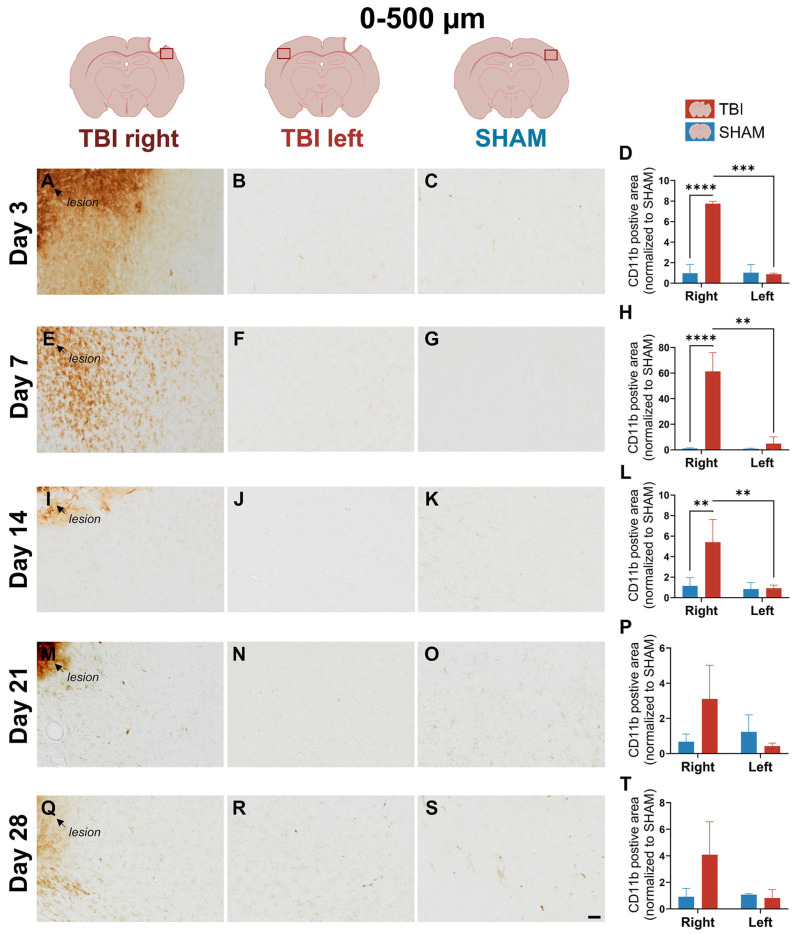
CD11b expression at 0–500 µm reveals early and sharply lateralized activation. Representative images and quantification of CD11b immunoreactivity in the peri-lesional cortex 0–500 µm from the lesion site across post-injury days 3 (**A**–**D**), 7 (**E**–**H**), 14 (**I**–**L**), 21 (**M**–**P**), and 28 (**Q**–**T**). CD11b staining is strongly elevated in TBI right at early timepoints, with minimal expression in SHAM and TBI left hemispheres. Quantification confirms significant group, hemisphere, and interaction effects from day 3 to day 14, indicating sharply lateralized microglial/macrophage activation. Statistical analyses were performed using two-way repeated-measures ANOVA with Holm–Sidak post hoc comparisons (SHAM left vs. TBI left; SHAM right vs. TBI right); hemispheric differences within TBI animals were assessed using paired *t*-tests with Holm–Sidak correction. Day 3, 7, 21, and 28: *n* = 3 (SHAM and TBI); day 14: *n* = 3 (SHAM) and 5 (TBI). Bars represent mean normalized values ± SD. The same statistical analyses were applied consistently to all quantified graphs in the figure. ** *p* < 0.01, *** *p* < 0.001, **** *p* < 0.0001. Scale bar = 50 µm, valid for all microphotographs. Brain illustrations created in BioRender. Hjæresen, S. (2025) (https://BioRender.com/dkneo0y, accessd 12 January 2026).

**Figure 4:**
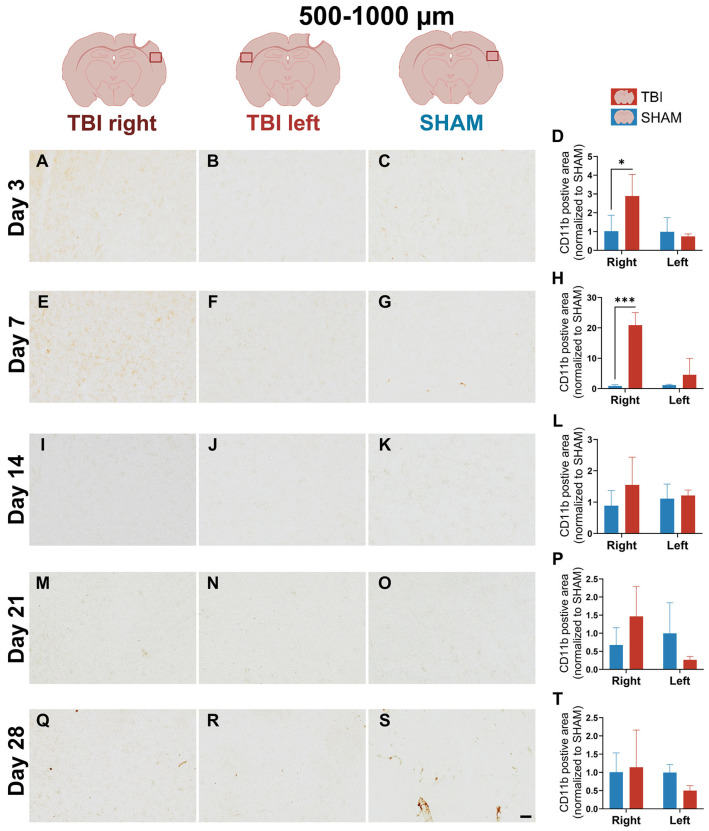
CD11b expression at 500–1000 µm shows attenuated and spatially constrained activation. Representative images and quantification of CD11b staining in the cortex 500–1000 µm from the lesion site across post-injury days 3 (**A**–**D**), 7 (**E**–**H**), 14 (**I**–**L**), 21 (**M**–**P**), and 28 (**Q**–**T**). At day 3, TBI right still shows elevated CD11b, though the intensity is lower and lateralization less pronounced than at 0–500 µm. Quantification reveals significant group, hemisphere, and interaction effects at early timepoints, but these resolve by day 14. Statistical analyses were performed using two-way repeated-measures ANOVA with Holm–Sidak post hoc comparisons (SHAM left vs. TBI left; SHAM right vs. TBI right); hemispheric differences within TBI animals were assessed using paired *t*-tests with Holm–Sidak correction. Day 3, 7, 21 and 28: *n* = 3 (SHAM and TBI); day 14: *n* = 3 (SHAM) and 5 (TBI). Bars show mean ± SD for each group and hemisphere. The same statistical analyses were applied consistently to all quantified graphs in the figure. * *p* < 0.025, *** *p* < 0.001. Scale bar = 50 µm, valid for all microphotographs. Brain illustrations created in BioRender. Hjæresen, S. (2025) (https://BioRender.com/dkneo0y, accessed 12 January 2026).

On day 3, strong and dense CD11b labeling was observed in the TBI right peri-lesional cortex (0–500 µm), with minimal staining in both SHAM right and TBI left hemispheres (Figure 3A–C). This marked asymmetry indicates a sharply localized increase in CD11b immunoreactivity. Two-way ANOVA confirmed significant main effects of group (*p* = 0.0023), hemisphere (*p* < 0.0001), and a group × hemisphere interaction (*p* < 0.0001) at 0–500 µm peri-lesional. Post hoc comparisons confirmed significantly elevated CD11b in TBI right compared to SHAM right (*p* < 0.0001), and a paired *t*-test confirmed lateralization within TBI animals (*TBI right > TBI left*: *p* = 0.0002) (Figure 3D). At 500–1000 µm, the CD11b-positive area was moderately increased in TBI right compared to the other groups (Figure 4A–C). Quantitatively, group (*p* = 0.0214), hemisphere (*p* = 0.0368), and interaction (*p* = 0.0347) effects were all significant, and TBI right remained elevated compared to SHAM right (*p* = 0.0027), indicating that a lateralized increase in CD11b immunoreactivity extended beyond the lesion core, though to a much lesser degree (Figure 4D).

At day 7, CD11b labeling in TBI right remained evident but less intense compared to day 3 (Figure 3E), while TBI left and SHAM hemispheres showed minimal staining (Figure 3F,G). Morphologically, CD11b+ cells in TBI right displayed irregular, partly stellate shapes and some with strong perinuclear labeling and less distinct process staining, consistent with a subacute activated phenotype (Supplementary Figure S1A (thick arrows)). Significant effects persisted at 0–500 µm perilesional group (*p* = 0.004), hemisphere (*p* = 0.001), and interaction (*p* = 0.001)). Post hoc tests confirmed that TBI right was elevated relative to SHAM right (*p* < 0.0001), and a paired *t*-test showed significant lateralization (*TBI right > TBI left*: *p* = 0.015) (Figure 3H). At 500–1000 µm, CD11b expression in TBI right also exceeded SHAM right (*p* = 0.0002), though the lateralization effect was weaker (Figure 4E–H).

At day 14, CD11b staining in TBI right appeared more diffuse and less intense (Figure 3I), suggesting a partial normalization of CD11b immunoreactivity. Quantification still showed significant effects at 0–500 µm peri-lesional (group (*p* = 0.034), hemisphere (*p* = 0.007), and interaction (*p* = 0.014)), with TBI right > SHAM right (*p* = 0.002), and persistent lateralization (*p* = 0.008) (Figure 3L). At 500–1000 µm peri-lesional, staining intensity appeared almost normalized across hemispheres, which matched the lack of statistical significance in the ANOVA and *t*-test (Figure 4I–L).

By days 21 and 28, CD11b expression had substantially declined in all groups, with no significant differences detected (Figure 3M–T and Figure 4M–T). Only faint labeling remained near the lesion, suggesting that CD11b immunoreactivity had largely returned toward baseline (Figure 3M).

### 3.2. IBA-1 Reveals Delayed Lateralization and Sustained Microglial Reactivity Through Day 21

To assess microglial dynamics, IBA-1 expression was quantified at 0–500 µm and 500–1000 µm from the lesion across the same five timepoints. Compared to CD11b, IBA-1 showed broader early response and delayed lateralization.

**Figure 5:**
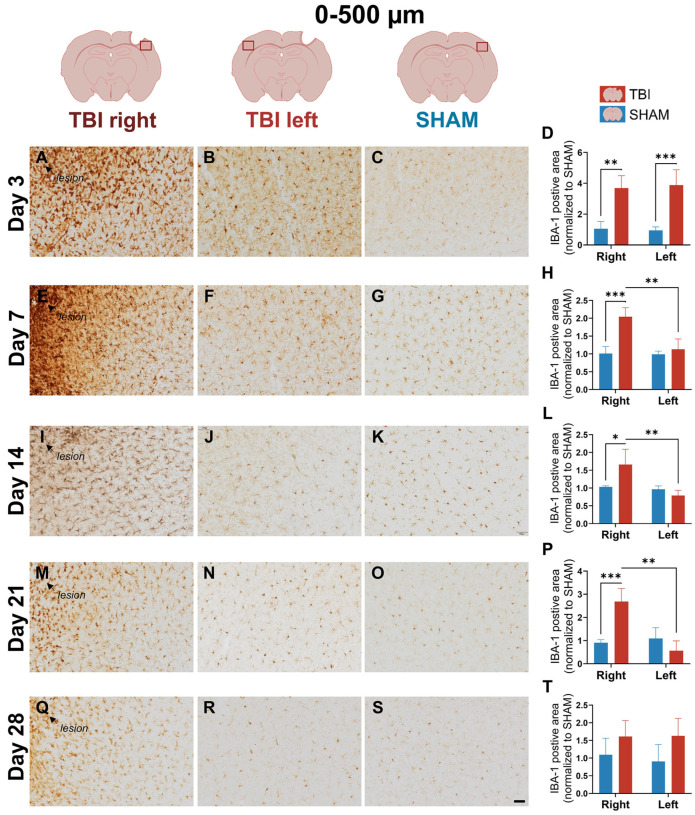
IBA-1 expression at 0–500 µm reveals delayed lateralization and sustained activation. Representative images and quantification of IBA-1 staining in the cortex 0–500 µm from the lesion across post-injury days 3 (**A**–**D**), 7 (**E**–**H**), 14 (**I**–**L**), 21 (**M**–**P**), and 28 (**Q**–**T**). At day 3, bilateral microglial activation is evident. From day 7 onward, labeling becomes lateralized to TBI right, persisting through day 21 and resolving by day 28. Statistical analyses were performed using two-way repeated-measures ANOVA with Holm–Sidak post hoc comparisons (SHAM left vs. TBI left; SHAM right vs. TBI right); hemispheric differences within TBI animals were assessed using paired *t*-tests with Holm–Sidak correction. Day 3: *n* = 3 (SHAM) and 4 (TBI); day 7: *n* = 3 (SHAM and TBI); day 14: *n* = 3 (SHAM) and 6 (TBI); day 21: *n* = 4 (SHAM) and 3 (TBI); day 28: *n* = 4 (SHAM and TBI). Bars = mean ± SD. The same statistical analyses were applied consistently to all quantified graphs in the figure. * *p* < 0.025, ** *p* < 0.01, *** *p* < 0.001. Scale bar = 50 µm, valid for all microphotographs. Brain illustrations created in BioRender. Hjæresen, S. (2025) (https://BioRender.com/dkneo0y, accessed 12 January 2026).

**Figure 6:**
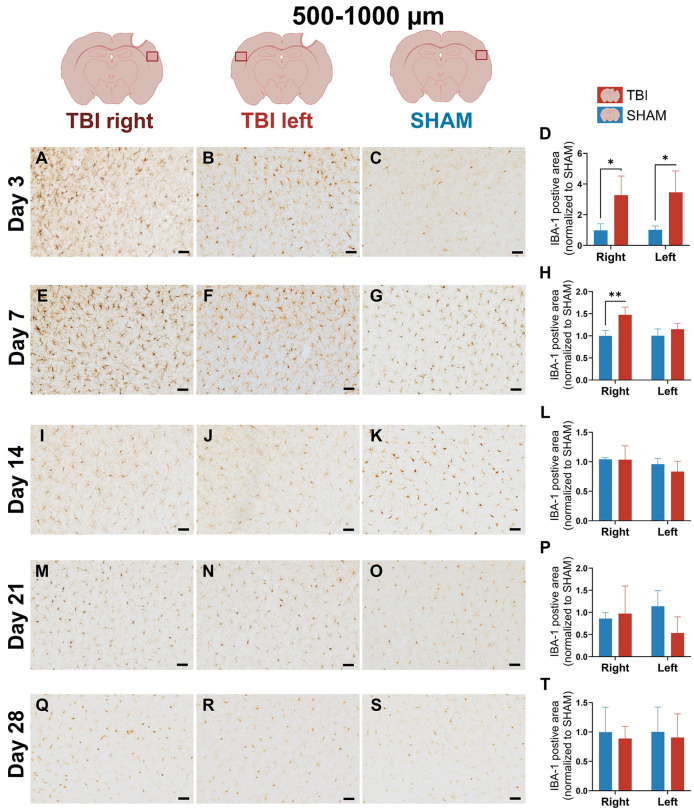
IBA-1 expression at 500–1000 µm demonstrates weaker, distal microglial reactivity. Images and quantification of IBA-1 staining at 500–1000 µm from the lesion site across post-injury on days 3 (**A**–**D**), 7 (**E**–**H**), 14 (**I**–**L**), 21 (**M**–**P**), and 28 (**Q**–**T**). Early activation (day 3) is moderate and bilateral, with delayed lateralization appearing from day 7. Activation in TBI right persists through day 21 but is less pronounced than in the proximal region. Quantitative analysis shows transient group and interaction effects, which were resolved by day 28. Statistical analyses were performed using two-way repeated-measures ANOVA with Holm–Sidak post hoc comparisons (SHAM left vs. TBI left; SHAM right vs. TBI right); hemispheric differences within TBI animals were assessed using paired *t*-tests with Holm–Sidak correction. Day 3: *n* = 3 (SHAM) and 4 (TBI); day 7: *n* = 3 (SHAM and TBI); day 14: *n* = 3 (SHAM) and 6 (TBI); day 21: *n* = 4 (SHAM) and 3 (TBI); day 28: *n* = 4 (SHAM and TBI). Bars represent mean ± SD. The same statistical analyses were applied consistently to all quantified graphs in the figure. * *p* < 0.025, ** *p* < 0.01. Scale bar = 50 µm, valid for all microphotographs. Brain illustrations created in BioRender. Hjæresen, S. (2025) (https://BioRender.com/dkneo0y, accessed 12 January 2026).

At day 3, IBA-1 labeling appeared bilaterally elevated in TBI animals, including the perilesional cortex in both TBI right and TBI left, while SHAM hemispheres showed less IBA-1 microglial staining (Figure 5A–C). This bihemispheric pattern was consistent with a generalized microglial marker expression immediately after injury. Quantification at 0–500 µm confirmed a significant group effect (*p* = 0.004), but no hemisphere or interaction effect, with both TBI hemispheres elevated over SHAM (*p* = 0.002 and *p* = 0.0008) (Figure 5D). At 500–1000 µm peri-lesional, IBA-1 labeling remained elevated in both TBI hemispheres, but to a lesser degree (Figure 6A–C). ANOVA showed a weaker group effect (*p* = 0.030), with no significant hemisphere or interaction effects, and no lateralization (Figure 6D).

At day 7, IBA-1 expression became more focused and intense in TBI right, while staining in TBI left and SHAM remained modest (Figure 5E–G). Morphologically, IBA-1^+^ microglia in TBI right displayed thicker and more prominent processes (Supplementary Figure S1D (thin arrows)) compared to SHAM, consistent with a subacute activated state. In TBI left, some microglia also showed more visible and elongated processes relative to SHAM, though this effect was less pronounced than ipsilaterally (Supplementary Figure S1E (thin arrows)). This visual shift suggests emerging lesion-side dominance. ANOVA at 0–500 µm revealed significant effects of group (*p* = 0.027), hemisphere (*p* = 0.0009), and group × hemisphere interaction (*p* = 0.001). Post hoc comparisons showed significantly elevated IBA-1 in TBI right vs. SHAM right (*p* = 0.0008), and a significant paired difference between TBI right and left (*p* = 0.008), confirming lateralization (Figure 5H). At 500–1000 µm peri-lesional, IBA-1 expression remained more diffuse, with a slightly elevated expression in the TBI brain (Figure 6E–G). Two-way ANOVA showed a significant interaction (*p* = 0.0007), and TBI right elevated over SHAM right (*p* = 0.008) meaning that lateralization remained statistically significant at 500–1000 µm peri-lesional, though it was weaker (*p* = 0.007) (Figure 6H).

On day 14, IBA-1 labeling remained strong but more diffuse than on day 7 in the TBI right hemisphere (Figure 5I), suggesting reduced but still elevated IBA-1 immunoreactivity. At 0–500 µm, ANOVA showed significant hemisphere (*p* = 0.007) and interaction (*p* = 0.014) effects. TBI right was still significantly higher than SHAM right (*p* = 0.012) and TBI left (*p* = 0.004), confirming ongoing lateralized IBA-1 immunoreactivity (Figure 5L). At 500–1000 µm pericontusiolesionalstaining appeared normalized across hemispheres (Figure 6I–K), with no significant ANOVA or paired effects (Figure 6L).

At day 21, IBA-1 labeling in TBI right (0–500 µm) remained detectably elevated (Figure 5M–O), with significant hemisphere (*p = *0.001) and interaction (*p* = 0.0006) effects; TBI right exceeded SHAM right (*p* = 0.0004) and TBI left (*p* = 0.002) (Figure 5P). At 500–1000 µm, no clear differences were observed (Figure 6M–P).

By day 28, IBA-1 labeling had largely returned to baseline across all groups (Figure 5Q–T and Figure 6Q–T). Only slightly higher expression persisted ipsilaterally, without statistical significance, consistent with normalization of the IBA-1 response similar to CD11b.

### 3.3. GFAP Expression Indicates Gradual, Prolonged Astrocytic GFAP Response That Extends Beyond Microglial Resolution

Astrocyte response was assessed via GFAP immunoreactivity. In contrast to the rapid, resolving profile of CD11b and IBA-1, GFAP showed a delayed onset and prolonged elevation in immunoreactivity.

**Figure 7:**
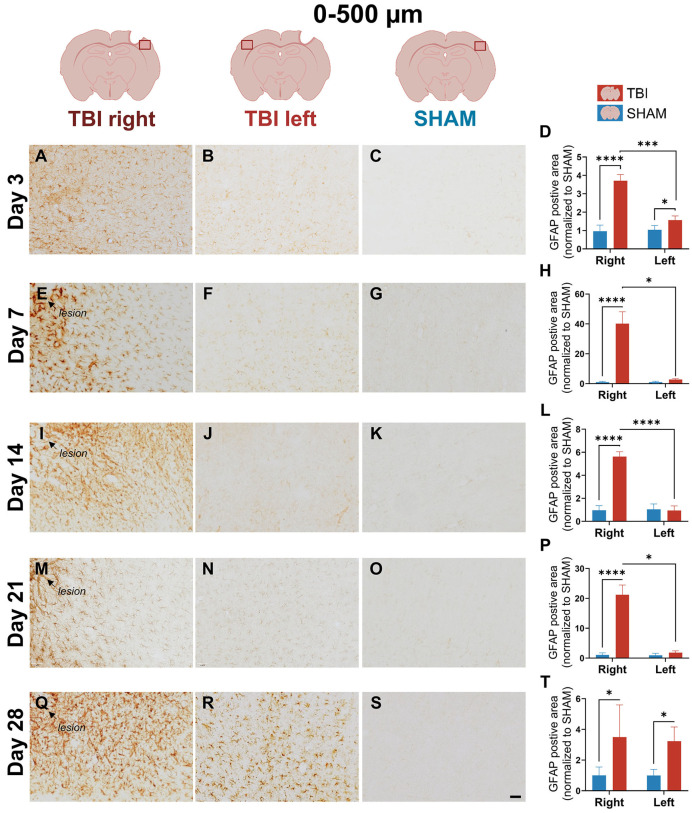
GFAP expression at 0–500 µm shows progressive and lateralized astrocytic activation. Representative GFAP staining and quantification in the peri-lesional cortex at 0–500 µm at post-injury days 3 (**A**–**D**), 7 (**E**–**H**), 14 (**I**–**L**), 21 (**M**–**P**), and 28 (**Q**–**T**). At day 3, TBI animals show a modest astrocytic response with a clear predominance in the injured hemisphere, increasing in intensity and spread by day 7. At day 21, activation persists, and by day 28, expression becomes bilaterally elevated. Statistical analyses were performed using two-way repeated-measures ANOVA with Holm–Sidak post hoc comparisons (SHAM left vs. TBI left; SHAM right vs. TBI right); hemispheric differences within TBI animals were assessed using paired *t*-tests with Holm–Sidak correction. Day 3: *n* = 4 (SHAM and TBI); Day 7 and 21: *n* = 3 (SHAM and TBI); Day 14: *n* = 3 (SHAM) and 6 (TBI); day 28: *n* = 4 (SHAM and TBI). Bars represent mean ± SD. The same statistical analyses were applied consistently to all quantified graphs in the figure. * *p* < 0.025, *** *p* < 0.001, **** *p* < 0.0001. Scale bar = 50 µm, valid for all microphotographs. Brain illustrations created in BioRender. Hjæresen, S. (2025) (https://BioRender.com/dkneo0y, accessed 12 January 2026).

At day 3, weak GFAP staining was observed in the peri-lesional cortex of TBI animals, with more prominent labeling in the TBI right hemisphere, while SHAM animals showed low GFAP immunoreactivity (Figure 7A–C). This indicates an early astrocytic response following injury. Quantification at 0–500 µm revealed significant effects of group and hemisphere as well as a group × hemisphere interaction (*p* = 0.0002). Post hoc comparisons confirmed increased GFAP immunoreactivity in TBI right compared to SHAM right (*p* < 0.0001), and a smaller but significant increase in TBI left compared to SHAM left (*p* = 0.0443) (Figure 7D). Within the TBI group, GFAP expression was significantly lateralized toward the injured hemisphere (*p* = 0.0007).

**Figure 8:**
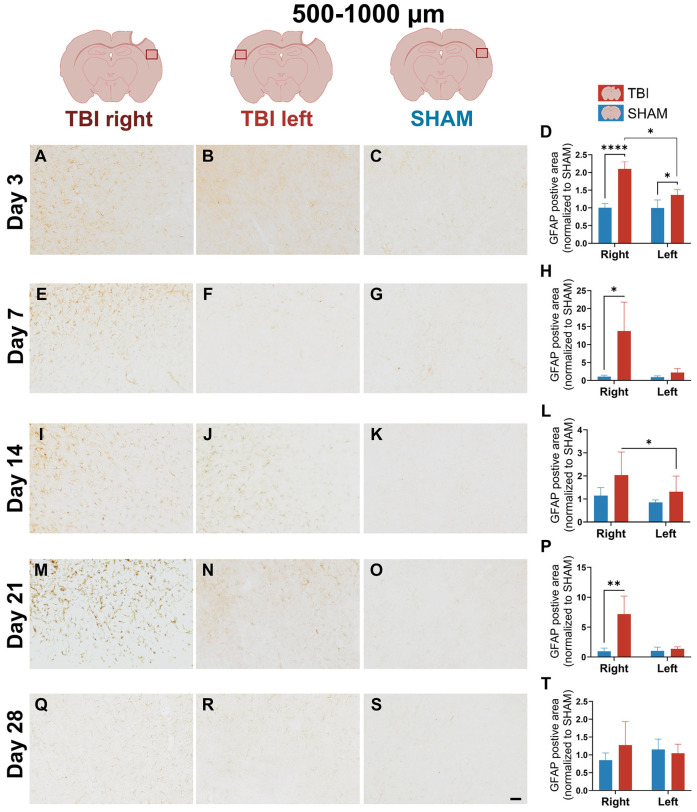
GFAP expression at 500–1000 µm indicates delayed and spatially variable astrocyte activation. Images and quantification of GFAP immunoreactivity at 500–1000 µm from the lesion site across post-injury days 3 (**A**–**D**), 7 (**E**–**H**), 14 (**I**–**L**), 21 (**M**–**P**), and 28 (**Q**–**T**). At day 3, GFAP immunoreactivity is low but detectable, with a predominance in the injured hemisphere. More pronounced increases are observed in TBI right from day 7 and persist until day 21. Quantitative data confirms time-dependent changes. Statistical analyses were performed using two-way repeated-measures ANOVA with Holm–Sidak post hoc comparisons (SHAM left vs. TBI left; SHAM right vs. TBI right); hemispheric differences within TBI animals were assessed using paired *t*-tests with Holm–Sidak correction. Day 3: *n* = 4 (SHAM and TBI); Day 7 and 21: *n* = 3 (SHAM and TBI); Day 14: *n* = 3 (SHAM) and 6 (TBI); day 28: *n* = 4 (SHAM and TBI). Bars = mean ± SD. The same statistical analyses were applied consistently to all quantified graphs in the figure. * *p* < 0.025, ** *p* < 0.01, **** *p* < 0.0001. Scale bar = 50 µm, valid for all microphotographs. Brain illustrations created in BioRender. Hjæresen, S. (2025) (https://BioRender.com/dkneo0y, accessed 12 January 2026).

At 500–1000 µm, GFAP immunoreactivity was also significantly increased in TBI animals. Two-way mixed ANOVA revealed significant main effects (*interaction*: *p* = 0.005; *hemisphere*: *p* = 0.0043; *group*: *p* = 0.0003) and post hoc comparisons showed elevated GFAP staining in both hemispheres (*TBI right* vs. *SHAM right*: *p* < 0.0001; *TBI left* vs.* SHAM left*: *p* = 0.0249). A significant hemispheric difference within the TBI group was also detected (*p* = 0.0121) (Figure 8A–D).

At day 7, GFAP staining intensified markedly in TBI right (Figure 7E) at 0–500 µm peri-lesion, with dense labeling not observed in other groups (Figure 7F,G). Morphologically, GFAP^+^ astrocytes in TBI right exhibited hypertrophic somata (Supplementary Figure S1G (thick arrows)) and thick, elongated processes with strong GFAP signal (Supplementary Figure S1G, (thin arrows)), consistent with a reactive phenotype, whereas astrocytes in TBI left and SHAM remained slender, with sparse labeling (Supplementary Figure S1H,I). This reflects emerging lesion-side astrocyte reactivity. ANOVA at 0–500 µm showed significant effects of group (*p* = 0.001), hemisphere (*p* = 0.001), and interaction (*p* = 0.001), with TBI right > SHAM right (*p* < 0.0001) and *TBI right > TBI left* (*p* = 0.014) (Figure 7H). At 500–1000 µm, effects were weaker (hemisphere: *p* = 0.044; interaction: *p* = 0.048), but lateralization persisted (*p* = 0.10) (Figure 8E–H).

At day 14, GFAP expression was dense and widespread in the perilesional cortex at 0–500 µm in TBI right, with astrocyte processes forming intense, interconnected networks (Figure 7I). In contrast, both TBI left and SHAM showed sparse GFAP expression (Figure 7H,J,K). This pattern indicates a robust astrocytic response, as reflected by an increased GFAP area in the injured hemisphere, which was supported by the ANOVA, which revealed significant effects of group (*p* = 0.003), hemisphere (*p* < 0.0001), and interaction (*p* < 0.0001). Post hoc tests confirmed TBI right > SHAM right (*p* < 0.0001) and *TBI right > TBI left* (*p* < 0.0001) (Figure 7L). At 500–1000 µm, visual inspection still revealed slightly more GFAP staining in TBI right, though it was less dense than closer to the lesion (Figure 8I). Although two-way ANOVA effects were not significant at this distance, the paired *t*-test showed significantly higher GFAP in TBI right vs. TBI left (*p* = 0.01), suggesting a lesion-biased but less spatially extensive astrocytic response at this stage (Figure 8L).

By day 21, GFAP remained elevated in TBI right, though staining appeared more diffuse compared to day 14 (Figure 7M). Interestingly, TBI left also exhibited visibly increased GFAP labeling, with scattered astrocytes showing hypertrophic morphology, supporting a bilateral, though still asymmetric, GFAP involvement (Figure 7N,O). At 0–500 µm, ANOVA showed significant group (*p* = 0.0003), hemisphere (*p* = 0.0009), and interaction (*p* = 0.0009) effects, with TBI right > SHAM right (*p* < 0.0001) and *TBI right > TBI left* (*p* = 0.012) (Figure 7P). At 500–1000 µm, GFAP staining in TBI right was visibly increased compared to all other groups (Figure 8M). TBI left also appeared slightly more reactive than SHAM, but with less intensity. ANOVA revealed significant effects of group (*p* = 0.0214), hemisphere (*p* = 0.0368), and interaction (*p* = 0.0347), and post hoc tests showed TBI right > SHAM right (*p* = 0.0027) (Figure 8P). These results support a delayed expansion of astrocytic reactivity, potentially indicating secondary spread of inflammation beyond the lesion core.

At day 28, GFAP labeling was visibly bilateral and more diffuse across both TBI hemispheres (Figure 7Q–S). Staining in TBI right remained higher than SHAM, but TBI left now showed almost comparable labeling, particularly at 0–500 µm. This pattern suggests a shift toward a more widespread and persistent GFAP signal. Statistically, only the group effect remained significant (*p* = 0.024), with no hemisphere or interaction effects, and the paired *t*-test between TBI right and left was non-significant (Figure 7T). At 500–1000 µm, GFAP signal was low and evenly distributed across groups, and all effects were non-significant (Figure 8Q–T).

### 3.4. Stable NeuN Immunoreactivity After Focal TBI

NeuN immunolabeling was used to assess neuronal soma density in the peri-lesional cortex at 0–500 µm and 500–1000 µm from the injury site across all post-injury timepoints.

**Figure 9:**
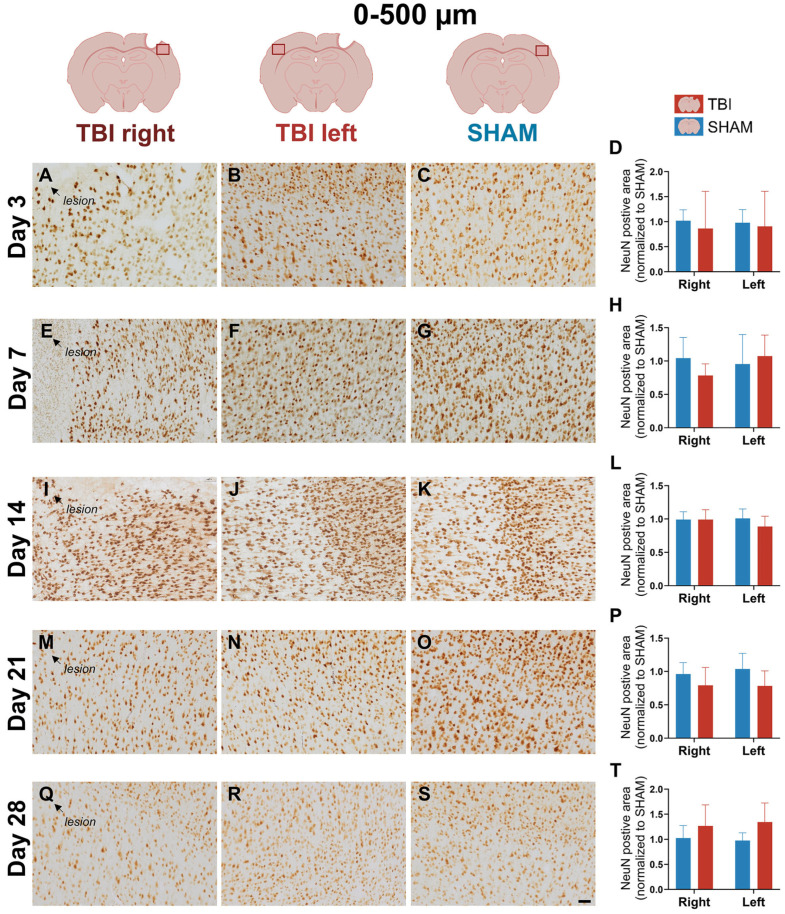
NeuN expression at 0–500 µm remains stable across timepoints, indicating preserved neuronal integrity. NeuN immunostaining and quantification in the peri-lesional region (0–500 µm) at post-injury days 3 (**A**–**D**), 7 (**E**–**H**), 14 (**I**–**L**), 21 (**M**–**P**), and 28 (**Q**–**T**). Visual inspection reveals consistent neuronal density and morphology across groups and timepoints. Quantitative analysis shows no significant group, hemisphere, or interaction effects, suggesting no overt neuronal loss. Statistical analyses were performed using two-way repeated-measures ANOVA with Holm–Sidak post hoc comparisons (SHAM left vs. TBI left; SHAM right vs. TBI right); hemispheric differences within TBI animals were assessed using paired *t*-tests with Holm–Sidak correction. Day 3 and 21: *n* = 4 (SHAM and TBI); day 7: *n* = 3 (SHAM and TBI); day 14: *n* = 3 (SHAM) and 6 (TBI); day 28: *n* = 4 (SHAM) and 5 (TBI). Bars = mean ± SD. The same statistical analyses were applied consistently to all quantified graphs in the figure. Scale bar = 50 µm, valid for all microphotographs. Brain illustrations created in BioRender. Hjæresen, S. (2025) (https://BioRender.com/dkneo0y, accessed 12 January 2026).

**Figure 10:**
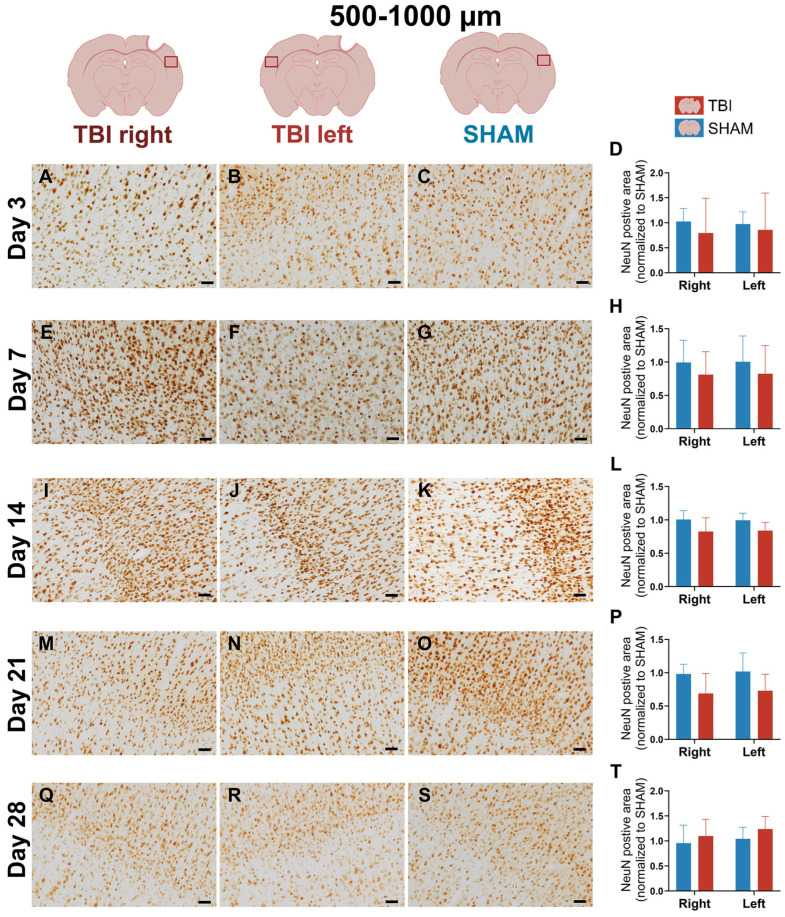
NeuN expression at 500–1000 µm confirms lack of neuronal loss in the distal cortex. NeuN immunostaining and quantification in the peri-lesional region at post-injury days 3 (**A**–**D**), 7 (**E**–**H**), 14 (**I**–**L**), 21 (**M**–**P**), and 28 (**Q**–**T**). NeuN expression appears uniform across groups and timepoints. Quantification reveals no significant differences in group, hemisphere, or interaction effects. These results support the conclusion that glial activation occurred without measurable neuronal loss in peri-lesional or distal cortical areas. Statistical analyses were performed using two-way repeated-measures ANOVA with Holm–Sidak post hoc comparisons (SHAM left vs. TBI left; SHAM right vs. TBI right); hemispheric differences within TBI animals were assessed using paired *t*-tests with Holm–Sidak correction. Day 3 and 21: *n* = 4 (SHAM and TBI); day 7: *n* = 3 (SHAM and TBI); day 14: *n* = 3 (SHAM) and 6 (TBI); day 18: *n* = 4 (SHAM) and 5 (TBI). Bars = mean ± SD. The same statistical analyses were applied consistently to all quantified graphs in the figure. Scale bar = 50 µm, valid for all microphotographs. Brain illustrations created in BioRender. Hjæresen, S. (2025) (https://BioRender.com/dkneo0y, accessed 12 January 2026).

Quantitative analysis revealed no significant effects of group, hemisphere, or their interaction at any timepoint (*two-way ANOVA*, all *p* > 0.05), indicating that the number of NeuN+ neurons remained stable following TBI (Figure 9 and Figure 10). Visual inspection of NeuN-stained sections supported these findings, with no overt signs of neuronal loss in the peri-lesional cortex at any timepoint (Figure 9 and Figure 10). Morphologically, NeuN^+^ neurons displayed preserved soma and dendritic outlines across all groups, with no detectable shrinkage or disorganization compared to SHAM (Supplementary Figure S1J–L). These observations suggest that our focal TBI model did not result in measurable neuronal loss within the sampled regions, despite the presence of sustained gliosis.

## 4. Discussion

The present study aimed to characterize the spatiotemporal dynamics of gliosis and neuronal preservation following focal TBI in the rat hindlimb sensorimotor cortex, with particular emphasis on differences between the ipsilateral and contralateral hemispheres and their potential relevance to the motor deficits previously reported in the same animals. In our aspiration model, neuronal loss was limited to the lesion core, with stable NeuN expression in the surrounding cortex. In contrast, astrocytes, microglia, and macrophages showed dynamic and regionally distinct responses. An early contralateral IBA-1^+^ microglial increase at day 3—despite the unilateral injury—is consistent with a more widespread response beyond the injured hemisphere. This was followed by sustained ipsilateral microglial activity and delayed, bilateral GFAP upregulation by day 28. These glial changes occurred in the absence of additional neuronal loss, consistent with the possibility that network-level remodeling rather than secondary neuronal degeneration contributes to functional outcomes. While the CD11b-associated myeloid response was transient and resolved during the subacute phase, IBA-1^+^ microglial and GFAP^+^ astrocytic responses persisted longer, indicating a more sustained involvement of resident glial populations.

### 4.1. Sequential Glial Responses in Focal Cortical Injury

#### 4.1.1. Early, Localized, and Transient CD11b^+^ Myeloid Activation in the Ipsilateral Cortex Following Focal Injury

The early peak in CD11b expression at day 3 may reflect acute myeloid activation involving resident microglia and/or infiltrating monocyte-derived cells. As CD11b is shared by both populations, our methods do not allow these to be distinguished; however, the sharply localized and transient peri-lesional signal—resolving by day 21—is consistent with reported myeloid responses following BBB disruption in moderate focal TBI models [16,17,18]. Although cytokines were not assessed here, prior studies show that CD11b^+^ cells are rapidly recruited to injury sites, where they participate in debris clearance and inflammatory signaling [18,19,20]. Similar early ipsilateral CD11b increases have been reported in focal CCI and LFPI models [16,17,19,21], aligning with the robust but spatially restricted CD11b response observed in our focal aspiration model at day 3, which persisted through days 7–14 and became non-significant by day 21.

Morphologically, CD11b^+^ cells displayed irregular, partly stellate shapes at day 7, consistent with subacutely reactive myeloid cells described in other rodent TBI models [14,16]. While phenotypic polarization was not assessed here, prior work demonstrates a temporal shift from pro- to anti-inflammatory myeloid states after TBI [16,18,19,20], suggesting that the observed CD11b peak reflects a continuum of activation states rather than a uniform population.

The magnitude and temporal profile of CD11b elevation are known to vary with injury severity, sex, and age. In female rats exposed to severe CCI, macrophage/microglial responses peak at 5–7 days, with anti-inflammatory markers rising around day 5 [20]. In contrast, the earlier and more spatially restricted ipsilateral CD11b response observed in our young adult male rats is consistent with a moderate focal injury and progressive resolution. Although C-C chemokine receptor type 2^+^ (CCR2^+^) CD11b^+^ monocytes have been linked to persistent neuroinflammation and worse outcomes in aged animals [18,22], we observed no evidence of sustained CD11b expression, suggesting that myeloid recruitment in this model is transient.

Altogether, the early CD11b response likely reflects an acute myeloid response that may include both resident microglia activation and recruitment of peripheral myeloid cells involved in acute surveillance, immune activation, and early remodeling.

Beyond their acute immune role, CD11b^+^ cells have increasingly been implicated in the initiation of glia-mediated remodeling after TBI. Although CD11b expression is transient in our model, early myeloid activation may influence subsequent microglial states and functions. Experimental studies indicate that activated microglia can form sustained contacts with neurons (“satellite microglia”), promote synaptic pruning, and alter local excitation–inhibition balance without overt neuronal loss [23]. Prolonged microglial activation has further been associated with synaptic degeneration, whereas microglial depletion can attenuate both structural and behavioral impairments [24]. Taken together, the early CD11b^+^ response observed here may reflect not only acute inflammatory activity, but also the initiation of glial plasticity processes that extend into the subacute phase.

#### 4.1.2. Early Bilateral and Sustained IBA-1^+^ Microglial Response Suggests Interhemispheric Involvement After Focal TBI

IBA-1 showed a prolonged increase up to day 21, consistent with sustained microglial reactivity over this interval and may be accompanied by changes in functional state, which were not directly assessed here. Similar temporal profiles have been reported in focal injury models, where IBA-1^+^ microglia typically increase during the first week and then gradually decline, although persistence up to day 28 has been observed in some CCI studies [20,25,26]. Injury severity and regional context further influence response duration [27,28]. In line with these reports, we observed ipsilateral IBA-1 upregulation from days 7–21 with normalization by day 28, consistent with a time-limited microglial response following moderate focal injury.

Morphologically, IBA-1^+^ microglia in the ipsilateral cortex displayed thickened processes at day 7, while contralateral cells showed subtler changes—features characteristic of subacute activation and regional heterogeneity [27,29,30].

A particularly interesting finding was the early increase in IBA-1 expression at day 3. While occasionally reported in focal CCI models [27,31], such early contralateral involvement is uncommon. In our case, it likely reflects both the dense interhemispheric connectivity of the hindlimb cortex and methodological sensitivity enabled by bilateral quantification. More severe injuries may mask such effects due to more localized damage. Recent evidence suggests that even focal injuries can provoke widespread immune responses via systemic or commissural pathways [32].

Notably, while IBA-1 became bilateral, CD11b remained confined ipsilaterally, consistent with the possibility that peripheral myeloid cells, if present, remain more spatially restricted, whereas resident microglial responses may extend more diffusely.

This bilateral IBA-1 pattern is consistent with the concept of glial network communication. Microglia can release cytokines such as IL-1α, TNF-α, and C1q to induce neurotoxic A1 astrocytes, or IL-10 and TGF-β to promote A2 phenotypes [7,33]. Although speculative, the temporal pattern—early IBA-1 activation followed by delayed GFAP elevation—may indicate a spreading glia–glia signaling cascade.

In addition to inflammation, sustained IBA-1 may reflect plasticity-promoting roles. The persistence of IBA-1 after CD11b resolution suggests a shift toward circuit remodeling. Subacute IBA-1 elevation has been linked to increased microglial–synapse contact, synaptic pruning, and modulation of dendritic spines and excitability [23]. These processes may be facilitated by earlier CD11b^+^ activation. Mechanistically, microglia have been shown to act via complement signaling, Brain-derived neurotrophic factor (BDNF) secretion, and ATP–adenosine pathways, influencing synaptogenesis and neuronal excitability [34]. Similar “bushy” microglial morphologies have been reported in stroke and diffuse TBI and are commonly associated with structural remodeling [35,36]. Together, these observations are consistent with a sequential microglial response in which early CD11b^+^ activity precedes later IBA-1^+^-associated remodeling.

In summary, the transient elevation and subsequent normalization of IBA-1 are consistent with a time-limited microglial response that may shift toward reparative or remodeling-associated functions, depending on injury context. Subacute IBA-1^+^ microglia have been reported to adopt IL-10^+^ and TGF-β^+^ phenotypes [37], and our findings align with a dynamic microglial response encompassing both immune and synapse-related processes.

#### 4.1.3. Delayed and Bilateral GFAP^+^ Astrogliosis Reflects Subacute-Chronic Glia Adaptions After Focal Injury

The GFAP response followed an early-subacute and prolonged course. In focal CCI models, GFAP increases modestly within the first 1–3 days but typically peaks after 4–7 days, reflecting astrocytic activation often associated with direct injury signals and sustained microglial reactivity [14,33]. Pericontusional astrocytosis are known to peaisithin the first week and gradually return toward baseline by day 28, although thalamic reactivity may persist [14]. In diffuse FPI, GFAP upregulation appears earlier and is more widespread, often persisting for weeks [38]. Severe or modified CCI models also report extended astrocytosis into later phases [25,26].

In our focal aspiration model, GFAP expression was already modestly increased bilaterally at day 3, but with a clear predominance in the injured hemisphere, increased sharply ipsilaterally by day 7, peaked at day 14, and showed delayed contralateral involvement by day 28. Thus, while early bilateral GFAP upregulation can be detected, the astrocytic response initially remains spatially biased toward the lesion side before evolving into a more widespread bilateral pattern. This aligns with reports of widespread and persistent astrogliosis in diffuse FPI models [38]. Importantly, the bilateral GFAP response observed at early and late time points likely reflects distinct biological processes. The shift from lateralized to bilateral signal fits with prior reports of astrocytic spread beyond the lesion. Morphologically, GFAP^+^ astrocytes at day 7 showed hypertrophic somata and thickened processes, consistent with a reactive phenotype, as also reported in focal CCI [30,39]. Remote astrocyte activation has also been described in interconnected regions (e.g., thalamus) and in human post-mortem studies, often influenced by survival time [14,40,41]. STAT3 signaling appears to mediate these transitions [32].

Variability across studies likely reflects injury severity, model type, and analysis methods. Moderate aspiration induces less edema and gliosis than severe CCI [20,25,26]. The hindlimb motor cortex’s strong interhemispheric connectivity may facilitate contralateral activation, and bilateral quantification enhances detection of subtle effects. Biological factors such as strain, age, and baseline inflammation further modulate glial dynamics.

Functionally, the temporal pattern—from an early, modest bilateral astrocytic response with predominantly ipsilateral expression to delayed bilateral spread—is consistent with potential dual astrocytic roles involving early containment near the lesion followed by later remodeling. Initial astrocytosis may support metabolic homeostasis and glial scarring, whereas sustained activity may reflect adaptive plasticity [42,43,44]. This progression parallels secondary injury processes, including oxidative stress, BBB disruption, and inflammation, and GFAP upregulation, may be shaped by early myeloid responses and NF-κB/JAK–STAT signaling cascades [7,14,16,31,42,45,46].

Beyond scar formation, delayed GFAP elevation has been associated with tissue recovery processes, including astrocytic upregulation of trophic factors (e.g., IGF-1, TGF-β1) and extracellular matrix components such as laminin and Chondroitin sulfcte proteoglycans (CSPGs), which may support angiogenesis and limit inflammation [47]. Although some studies suggest a shift toward reparative astrocytic programs at later stages, phenotype-specific markers were not assessed here [48,49].

GFAP^+^ astrocytes have also been implicated in synaptogenesis and network remodeling through the release of synaptogenic factors (e.g., thrombospondins, hevin, D-serine, cholesterol, BDNF) and through homeostatic regulation of glutamate and potassium via EAATs and Kir4.1 channels [48,50,51,52]. Together, the bilateral GFAP profile observed here is consistent with a combination of scarring and circuit-stabilizing roles.

In summary, the sequence of early CD11b recruitment, sustained IBA-1 elevation, and delayed GFAP upregulation captures a stepwise evolution of secondary injury, with the early, predominantly ipsilateral astrocytic response followed by a later, more diffuse bilateral engagement consistent with broader signaling and remodeling processes during later stages.

#### 4.1.4. Stable NeuN Expression with Potential Subcellular Dysfunction After Focal Injury

NeuN expression remained stable across all timepoints, indicating preserved neuronal density in the pericontusional cortex. Morphologically, NeuN^+^ neurons appeared intact, with well-preserved somata and some dendritic outlines across groups, and no overt shrinkage or disorganization compared to SHAM. This agrees with previous findings that moderate focal injury can maintain cortical NeuN^+^ cell numbers despite reactive glial cells and that reduced NeuN immunoreactivity does not necessarily indicate neuronal death but may reflect reversible protein modulation [53,54].

However, NeuN does not reflect functional or subcellular integrity. Axonal injury, synaptic disruption, or metabolic dysfunction may occur independently of NeuN loss [53,55]. Thus, the absence of NeuN reduction does not exclude subtle neuronal impairments that could contribute to motor deficits. Future studies assessing synaptic proteins, axonal markers, or mitochondrial function would clarify neuronal status beyond soma preservation.

Our findings resemble those in other moderate focal models, where NeuN^+^ cortical populations remain intact while hippocampal or subcortical regions are more vulnerable [56,57]. In contrast, severe CCI and diffuse injuries show pronounced NeuN loss in the cortex and hippocampus [28,55,58,59,60], underscoring the influence of injury severity and location.

Despite stable neuronal counts, the pronounced glial responses suggest ongoing network remodeling. As stated above, reactive microglia and astrocytes modulate synaptic structure, plasticity, and excitability in the absence of neuronal death [18,22,23,24].

In summary, NeuN stability supports neuronal survival, but functional or molecular impairments cannot be excluded. The observed glial and monocyte responses likely reflect adaptive remodeling rather than overt degeneration in our moderate focal model.

### 4.2. Glial and Neuroendocrine Mechanisms of Persistent Motor Deficits After TBI

While primary motor deficits after TBI arise from the immediate loss of cortical neurons and the disruption of corticospinal pathways, accumulating evidence implicates neuroinflammation, network remodeling, and neuroendocrine signaling as important secondary contributors. Experimental studies show that dampening inflammatory pathways—through microglial suppression, inflammasome inhibition, or cytokine blockade—can improve motor recovery across multiple TBI models [46,61,62,63,64,65,66]. Even aspiration lesions, though less studied, elicit robust glial responses linked to behavioral changes [67].

In our model, the sharply lateralized CD11b response at day 3 coincided with early contralateral hindlimb asymmetry, while sustained ipsilateral IBA-1 activation and a progression from early, predominantly ipsilateral to later more pronounced bilateral GFAP upregulation matched the later emergence of more generalized motor dysfunction [6]. Stable NeuN counts in the peri-lesional cortex are consistent with the possibility that, beyond the primary neuronal loss confined to the lesion core, persistent motor impairments may additionally reflect network-level glial remodeling rather than overt secondary neuronal degeneration. However, as no animal-level correlation between histology and behavior was performed, these relationships remain associative.

Taken together, the staggered time courses of early myeloid/microglial activation (CD11b/IBA-1) and the temporally progressive astrocytic response (GFAP) are consistent with a transition from acute immune surveillance and debris handling toward longer-term plasticity and homeostatic remodeling. Through synapse–glia signaling, microglia and astrocytes can modulate synaptic connectivity, excitability, and extracellular matrix composition without inducing neuronal loss, thereby potentially influencing both repair processes and persistent functional deficits.

Notably, only IBA-1 and GFAP responses extended bilaterally, whereas CD11b remained spatially confined. This pattern is consistent with the possibility that peripheral myeloid contributions are restricted to the injured hemisphere, while resident glia—particularly microglia and astrocytes—engage in interhemispheric or systemic signaling. In line with this, we previously reported bilateral remodeling of hindlimb muscle extracellular matrix following unilateral cortical injury [68]. However, the mechanisms underlying bilateral activation (e.g., commissural versus humoral pathways) were not directly assessed here and therefore remain speculative.

As stated above, sustained glial activation beyond the lesion site may contribute to circuit dysfunction. Microglia can prune synapses via complement signaling, while astrocytes influence excitability through glutamate and ATP release and regulation of GLT-1 and Kir4.1 channels [34,36,51,69,70,71,72]. Persistent glial activity may also constrain axonal sprouting and synaptic plasticity through scar-associated extracellular matrix changes, such as CSPG accumulation. Thus, even in the absence of NeuN loss, disrupted transport or mitochondrial dysfunction may persist and contribute to ongoing motor deficits [47,69].

Neuroendocrine signaling may further modulate these processes. Cortical injury alters hypothalamic–pituitary output, and hormones such as vasopressin, oxytocin, β-endorphin, and glucocorticoids influence both inflammation and behavior [73,74,75,76,77]. Prior studies, including work from our group, demonstrate that unilateral motor cortex injury can disrupt posture and locomotion via pituitary-dependent modulation of glial activity [75,78,79]. CCI-induced hypothalamic activation has likewise been linked to long-term behavioral impairments, likely through HPA axis dysregulation and hormone-driven glial reorganization [76,77].

Together, these findings suggest that neuroendocrine–immune crosstalk may contribute to the bilateral microglial and astrocytic responses observed here, despite a unilateral lesion. Within this framework, the early bilateral IBA-1 increase may help explain the previously reported contralateral motor asymmetry in this model, consistent with the possibility that microglial engagement can precede and amplify functional impairment before astrocytic activation becomes more pronounced and widespread.

### 4.3. Limitations

This study has several limitations. Group sizes were relatively small for some markers, and minor variability may arise from differences in staining, imaging, and manual threshold selection for a subset of images (day 28), despite the use of a consistent ImageJ analysis pipeline and normalization to time-matched SHAM controls. Quantification was restricted to the peri-lesional cortex and based on percentage area of DAB signal, which does not resolve glial subtypes or capture axonal or synaptic pathology. NeuN reflects neuronal soma density but not neuronal function, axonal integrity, or synaptic loss, so subtle neuronal impairments may be underestimated. Similarly, CD11b, IBA-1, and GFAP indicate general glial activation without distinguishing functional phenotypes. Only male rats were included in this study; therefore, potential sex-specific differences in glial or neuronal responses to TBI were not assessed and should be addressed in future studies. Finally, no direct correlation analysis between histological measures and behavioral outcomes was performed; thus, the link between glial activation and motor impairment remains associative rather than causal.

## 5. Conclusions

This study demonstrates that a highly focal cortical injury elicits a sequential and cell-type-specific glial response, with early ipsilateral CD11b^+^ myeloid activation followed by prolonged IBA-1^+^ microglial activation and a progression from early lesion-biased to later bilateral GFAP^+^ astrocytic reactivity. While CD11b remained spatially confined, the bilateral spread of resident glial markers suggests broader network engagement independent of overt neuronal loss. The glial dynamics parallel known motor deficits in this model and may reflect a combination of local injury responses and systemic modulation. These findings highlight the potential role of glial remodeling in shaping functional outcomes after focal TBI.

## Data Availability

The data presented in this study are available on request from the corresponding author.

## Supplementary Materials

The following supporting information can be downloaded at https://www.mdpi.com/article/10.3390/life16010142/s1: Figure S1: Glial and neuronal morphology at 0–500 µm reveals reactive phenotypes in the peri-lesional cortex; Table S1: Primary and secondary antibodies used for IHC; Table S2: Analysis parameters for quantification in ImageJ; Table S3: Overview of experimental groups and animal numbers at each timepoint.

## Abbreviations

The following abbreviations are used in this manuscript:

TBI	Traumatic brain injury
BBB	Blood–brain barrier
HL-PA	Hindlimb postural asymmetry
DAMP	Damage-associated molecular pattern
NF-κβ	Nuclear factor kappa-light-chain-enhancer of activated B cells
IL	Interleukins
TNF-α	Tumor necrosis factor alpha
FPI	Fluid percussion injury
GFAP	Glial fibrillary acidic protein
CCI	Controlled cortical impact
CD11b	Cluster of differentiation 11b
IBA-1	Ionized calcium-binding adapter molecule 1
i.p.	Intraperitoneally
PBS	Phosphate-buffered saline
NeuN	Neuronal nuclei antige
BSA	Bovine serum albumin
NGS	Normal goat serum
RT	Room temperature
DAB	3,3′-diaminobenzidine
LFPI	Lateral fluid percussion injury
CCR2	C-C chemokine receptor type 2
BDNF	Brain-derived neurotrophic factor
CSPG	Chondroitin sulfate proteoglycan
TGF-β	Transforming growth factor beta
JAK	Janus kinase
STAT	Signal transducer and activator of transcription
HMGB1	High mobility group box 1
NLRP3	NOD-, LRR- and pyrin domain-containing protein 3
AVP	Vasopressin
OXT	Oxytocin
