# Supplementary material for: Temporal and Spatial Patterns of Glial Activation After Unilateral Cortical Injury in Rats
Source: Life (Basel). 2026 Jan 15;16(1):142. doi: 10.3390/life16010142 (PMC12843123; doi:10.3390/life16010142)
Supplement: Supplementary file 1 [file life-16-00142-s001.zip › life-4040683-supplementary.pdf]

Supplementary materials:

Supplementary Table S1. Primary and secondary antibodies used for IHC

Specific marker	Celle type	Primary antibody	Secondary antibody
CD11b (OX42)	Macrophages and pro-inflammatory microglia	Mouse anti Cd11b, 1:1000, BIORAD (MCA275G)	Goat anti mouse biotinylated, 1:2000, Sigma (SAB4600005)
IBA-1	Microglia	Rabbit anti IBA-1, 1:500, Abcam (ab178846)	Goat anti rabbit biotinylated, 1:5000, Sigma (SAB4600007)
GFAP	Astrocytes	Rabbit anti GFAP, 1:1000, Sigma-Aldrich (G9269)	Goat anti rabbit biotinylated, 1:5000, Sigma (SAB4600007)
NeuN	Mature neurons	Rabbit anti NeuN; 1:500, Millipore (ABN78)	Goat anti rabbit biotinylated, 1:5000, Sigma (SAB4600007)

Supplementary Table S2. Analysis parameters for quantification in ImageJ

Antibody	No. of timepoints analysed	Rolling ball radius	Lower threshold	Upper threshold
CD11b	7	300	0	237
IBA-1	6	300	0	215
GFAP	6	300	0	230
NeuN	6	300	0	235

Supplementary Table S3. Overview of experimental groups and animal numbers at each timepoint.

Timepoint (days post injury)	SHAM rats (no.)	TBI rats (no.)
3	4 (3 for CD11b)	4 (3 for CD11b)
7	3	3
14	3	6
21	4 (3 for GFAP)	4 (3 for IBA-1 and GFAP)
28	4 (3 for CD11b)	5 (3 for CD11b and 4 for IBA-1)

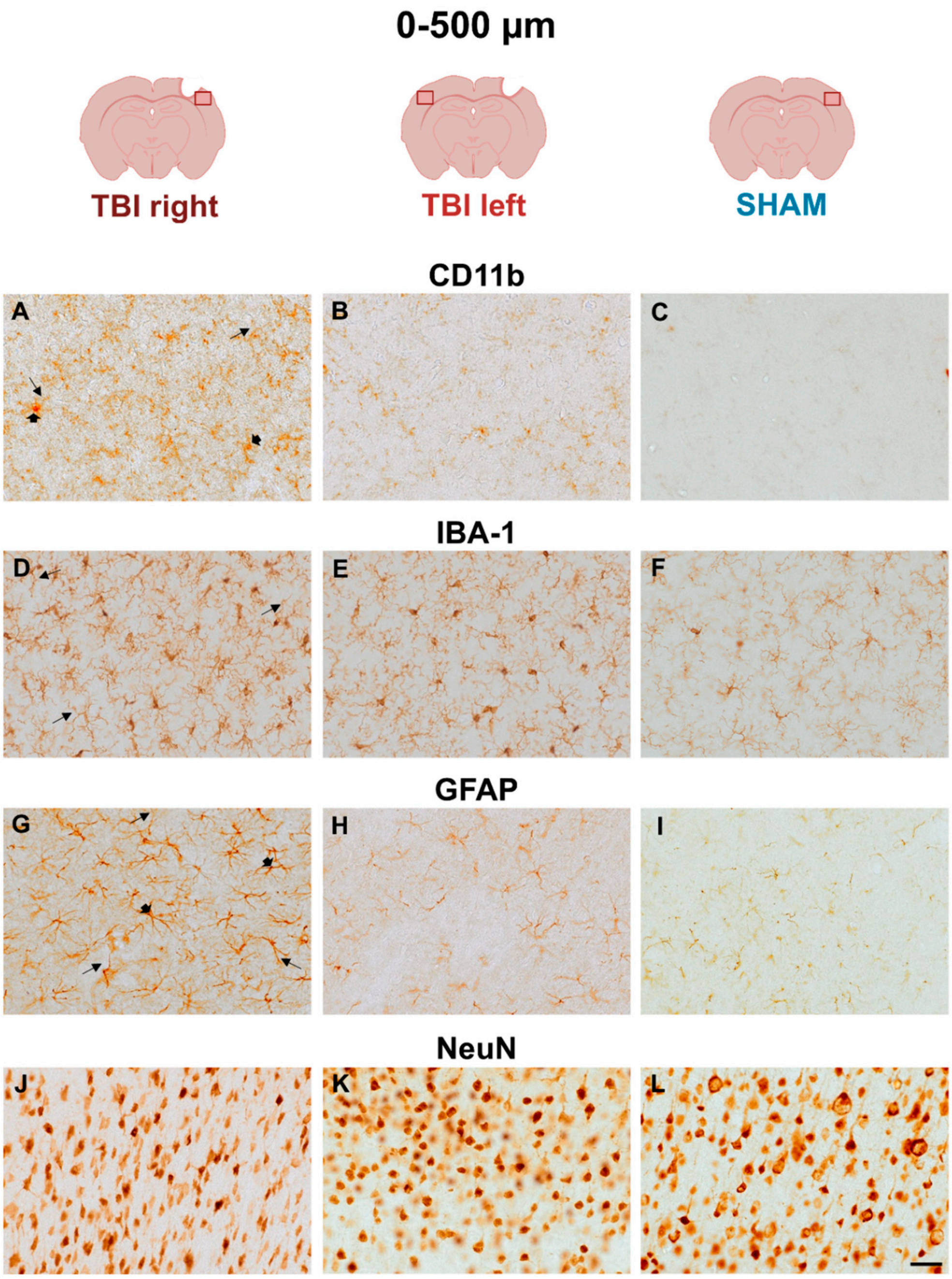

Supplementary Figure S1. Glial and neuronal morphology at 0–500 µm reveals reactive phenotypes in peri-lesional cortex.

Representative immunohistochemical images showing CD11b$^+$, IBA-1$^+$, GFAP$^+$, and NeuN$^+$ cells within 0–500 µm from the lesion 7 days post-injury at higher magnification. CD11b$^+$ cells in TBI right displayed irregular, partly stellate shapes with strong perinuclear labeling and less distinct process staining, consistent with a subacute activated phenotype (A, thick arrows). IBA-1$^+$ microglia in TBI right exhibited thicker and more prominent processes compared to SHAM, whereas TBI left showed a milder activation pattern (D–F, thin arrows). GFAP$^+$ astrocytes in TBI right presented hypertrophic somata and elongated, thick processes with intense labeling, characteristic of a reactive state, while astrocytes in TBI left and SHAM remained slender with sparse GFAP expression (G–I, arrows). NeuN$^+$ neurons displayed preserved somata and dendritic organization across groups, with no evidence of neuronal shrinkage or disarray relative to SHAM (J–L). These observations indicate marked glial activation without detectable neuronal loss in peri-lesional cortex at 0–500 µm. Scale bar = 50 µm.
